# Deep feature batch correction using ComBat for machine learning applications in computational pathology

**DOI:** 10.1016/j.jpi.2024.100396

**Published:** 2024-09-12

**Authors:** Pierre Murchan, Pilib Ó Broin, Anne-Marie Baird, Orla Sheils, Stephen P Finn

**Affiliations:** a Department of Histopathology and Morbid Anatomy, Trinity Translational Medicine Institute, Trinity College Dublin, Dublin D08 W9RT, Ireland; b The SFI Centre for Research Training in Genomics Data Science, Dublin, Ireland; c School of Mathematical & Statistical Sciences, University of Galway, Galway H91 TK33, Ireland; d Department of Histopathology, St. James's Hospital, James's Street, Dublin D08 X4RX, Ireland; e School of Medicine, Trinity Translational Medicine Institute, Trinity College Dublin, Dublin D02 A440, Ireland

**Keywords:** Computational pathology, Artificial intelligence, Batch effects, The Cancer Genome Atlas (TCGA), Histopathology

## Abstract

**Background:**

Developing artificial intelligence (AI) models for digital pathology requires large datasets from multiple sources. However, without careful implementation, AI models risk learning confounding site-specific features in datasets instead of clinically relevant information, leading to overestimated performance, poor generalizability to real-world data, and potential misdiagnosis.

**Methods:**

Whole-slide images (WSIs) from The Cancer Genome Atlas (TCGA) colon (COAD), and stomach adenocarcinoma datasets were selected for inclusion in this study. Patch embeddings were obtained using three feature extraction models, followed by ComBat harmonization. Attention-based multiple instance learning models were trained to predict tissue-source site (TSS), as well as clinical and genetic attributes, using raw, Macenko normalized, and Combat-harmonized patch embeddings.

**Results:**

TSS prediction achieved high accuracy (AUROC > 0.95) with all three feature extraction models. ComBat harmonization significantly reduced the AUROC for TSS prediction, with mean AUROCs dropping to approximately 0.5 for most models, indicating successful mitigation of batch effects (e.g., CCL-ResNet50 in TCGA-COAD: Pre-ComBat AUROC = 0.960, Post-ComBat AUROC = 0.506, *p <* 0.001). Clinical attributes associated with TSS, such as race and treatment response, showed decreased predictability post-harmonization. Notably, the prediction of genetic features like MSI status remained robust after harmonization (e.g., MSI in TCGA-COAD: Pre-ComBat AUROC = 0.667, Post-ComBat AUROC = 0.669, *p*=0.952), indicating the preservation of true histological signals.

**Conclusion:**

ComBat harmonization of deep learning-derived histology features effectively reduces the risk of AI models learning confounding features in WSIs, ensuring more reliable performance estimates. This approach is promising for the integration of large-scale digital pathology datasets.

## Highlights

• ComBat harmonization can be used to effectively reduce batch effects in deep learning-derived histology features.
• Key histological information in deep learning-derived features is retained after ComBat harmonization such that clinical attributes remain predictable.
• This approach may help to improve the integration of large-scale digital pathology datasets by reducing the risk of overfitting to confounding features.

## Introduction

**Fig. 1:**
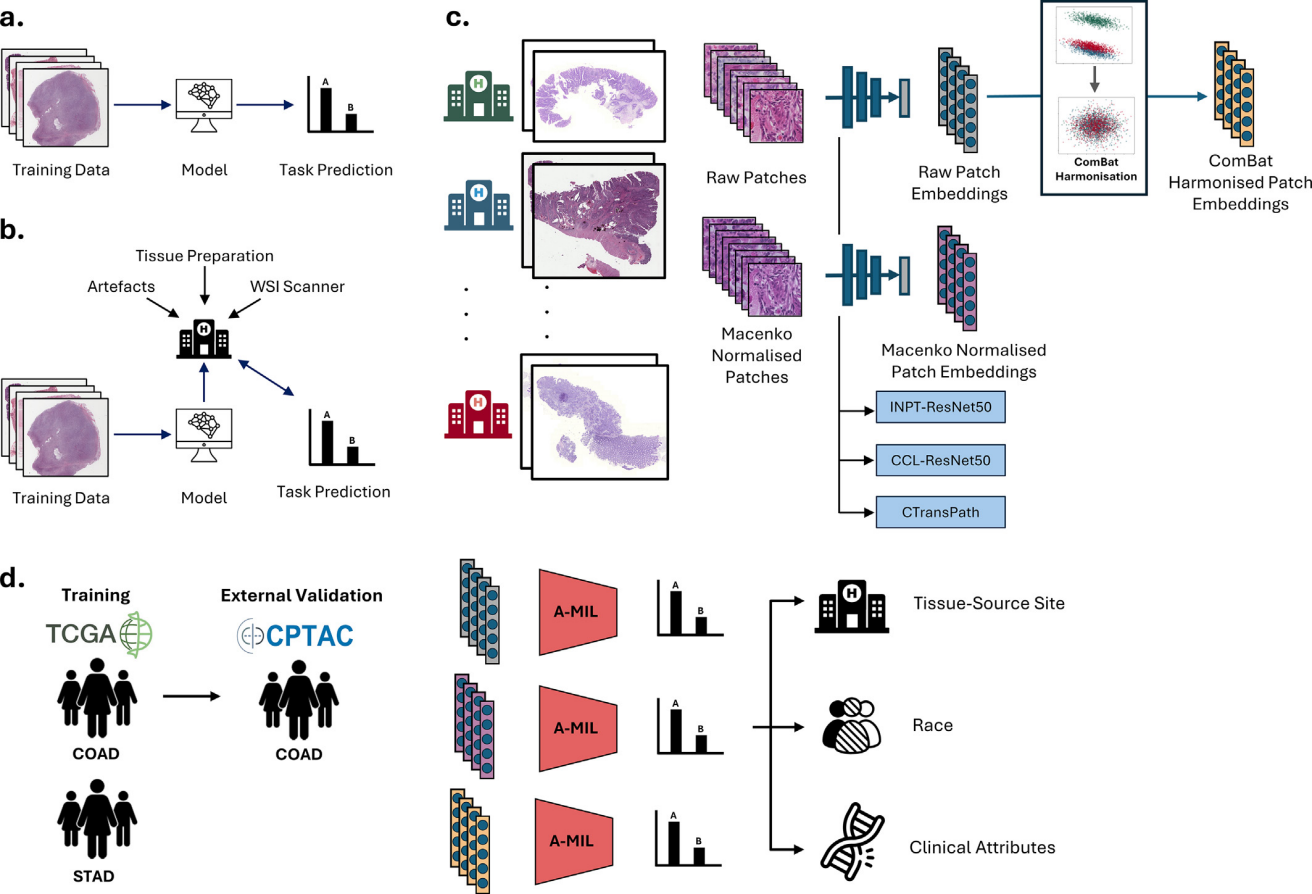
Study design. (a) Expected machine learning training paradigm in computational pathology. The machine learning model learns to predict a task of interest from histological features in WSI training data. (b) Potential machine learning training paradigm in computational pathology. The machine learning model learns confounding features in WSIs that are associated with both the task of interest and the site at which the tissue was prepared. (c) Preprocessing of WSIs. WSIs originated from multiple different TSSs. WSIs were patched and background and blurry patches were removed. Feature extraction models, INPT-ResNet50, CCL-ResNet50, and CTransPath, were used to obtain embeddings from both raw and Macenko normalized patches. Raw patches were subject to ComBat harmonization to mitigate TSS-specific batch effects. (d) A-MIL models were trained to predict TSS, race and clinical attributes using raw, Macenko normalized, and ComBat harmonized patch embeddings. Data from TCGA-COAD and TCGA-STAD cohorts were used to train A-MIL models. Models trained to predict clinical attributes on TCGA-COAD were externally validated on CPTAC-COAD.

High-quality, large-scale, and diverse datasets are crucial for developing machine learning models for healthcare applications. However, curating such datasets with reliable and robust annotations is challenging across medical domains. In the field of digital pathology, The Cancer Genome Atlas (TCGA) has emerged as a pivotal resource for researchers, providing over 20,000 whole-slide images (WSIs) accompanied by genomic and clinical data.^1^ However, without careful implementation, artificial intelligence (AI) models risk learning confounding features in datasets such as TCGA instead of clinically-relevant information (Fig. 1a–b).^2^, ^3^, ^4^ Digital pathology datasets, in particular, are susceptible to technical biases introduced by variations in tissue staining, WSI scanning, and image compression protocols.^5^, ^6^, ^7^ Such biases tend to vary more across clinical sites at which tumor tissue is processed and slides are digitized, referred to as tissue–source site or TSS from this point forward, rather than within sites.^5^^,^^8^ Previous research has shown that patient outcomes and genomic features can be associated with TSSs within the TCGA dataset.^9^ This can introduce confounding factors for AI models such that models learn shortcuts through site-specific features instead of true histological features. Overfitting to these phenotype-irrelevant features can lead to inflated model performance on internal validation sets and models which may not generalize to real-world settings.^10^ (See Fig. 1:.)

Strategies employed to mitigate site-specific biases in WSIs include stain normalization, which adjusts image colors to fit to a single distribution,^11^, ^12^, ^13^, ^14^ and color augmentation, where artificial variations in color are introduced at the time of training.^15^ However, these methods are ineffective at preventing models from learning TSS-specific features and omitting these steps from computational pathology pipelines has been shown to have no performance benefit on downstream slide-level classification performance when using modern feature extractor models.^8^^,^^16^ Site-stratified cross-validation, a bias mitigation strategy where all WSIs belonging to the same site are contained in the same fold of a cross-validation experiment, has been proposed as an alternative to color normalization and augmentation.^8^ Whereas this approach reduces the risk of overestimating model performance on internal datasets, it does not prevent a model from learning and relying on batch effects in WSIs during the training process. Therefore, new strategies are needed to mitigate the risk of AI models learning potentially confounding features in digital pathology datasets.

In recent years, computational pathology researchers have shifted away from end-to-end training of deep learning models in favor of applying pretrained feature extractors to embed the information contained in WSI patches into lower-dimensional numerical feature vectors.^17^ A second model is then typically trained to aggregate patch-level embeddings and predict the target attribute of interest. Whereas early studies relied on convolutional neural networks (CNNs) pretrained on Imagenet to extract features from WSI patches, recent advancements in deep learning have enabled researchers to make use of models pretrained on histology data using self-supervised learning (SSL).^18^, ^19^, ^20^, ^21^, ^22^ SSL enables a model to learn rich, domain-specific, feature representations, or embeddings, from unlabeled data. Whereas domain-specific pretraining has been shown to improve the performance, generalizability, and fairness of AI models in computational pathology, its impact on enabling downstream classification models to learn site-specific information from WSIs remains unclear.^23^^,^^24^

Bias in training data has been extensively characterized in medical applications of machine learning.^25^, ^26^, ^27^ Batch correction is a widely used technique across various domains to address systematic biases and confounding factors that can affect the analysis of high-dimensional datasets.^28^, ^29^, ^30^ Although the concept of batch correction initially gained prominence in bioinformatics, particularly in the context of RNA sequencing data, it has also been adopted by other fields such as radiomics.^31^, ^32^, ^33^ One widely used method for correcting batch effects in high-dimensional data is Compressed Batch Adjustment of Batch Effects (ComBat) which was originally developed for gene expression data.^34^ ComBat aims to model the sources of batch effects using an empirical Bayes framework and adjusts the data to remove unwanted variations while preserving the biological or clinical signal of interest.

In this study, we explore the impact of model architecture and pretraining on extracting TSS-specific features from WSIs. Furthermore, we demonstrate how ComBat harmonization of deep features outperforms standard Macenko color normalization at preventing models from learning potentially confounding batch effects in digital pathology datasets.

## Methods

### Patient cohort and data

Publicly available datasets from TCGA were used as the primary cohort for this study, specifically, the colon (COAD) and stomach adenocarcinoma (STAD) datasets. These were selected as these cohorts have a number of clinical features that have been previously reported as predictable from WSIs using deep learning models.^35^^,^^36^ TCGA diagnostic WSIs from formalin-fixed paraffin-embedded tissues were used for this study. External validation of models trained on the TCGA-COAD cohort was carried out on the Clinical Proteomic Tumor Analysis Consortium (CPTAC) COAD dataset.^37^ WSIs for the CPTAC-COAD dataset are publicly available for download from The Cancer Imaging Archive.^38^ Corresponding clinical data for TCGA and CPTAC datasets were retrieved from cBioPortal.^39^

#### Clinical attribute inclusion criteria

The association of attributes such as race (Clinical Data Element ID: 2192199), primary therapy outcome success (Clinical Data Element ID: 2786727) and mutated genes with TSSs were evaluated in order to identify potential features that could be predictable through confounding batch effects. Attributes were considered for inclusion in the study provided they had a minimum of 30 samples in the minority class. A minimum of 30 samples was selected to ensure sufficient data for training and evaluation in cross-validation experiments. The association of clinical attributes with TSS was evaluated using chi-squared tests with significance determined using a false-discovery rate (FDR) of 0.05 with the Benjamini–Hochberg method applied individually to each cancer subtype. Additionally, a number of biomarkers that have previously been shown to be predictable from WSIs were included in order to assess if these biomarkers remain predictable after applying ComBat harmonization.^35^^,^^36^^,^^40^ For example, microsatellite instability (MSI), an approved biomarker for immunotherapy treatment in colorectal cancer, has been extensively validated as predictable from WSIs.^41^ Furthermore, MSI in colorectal cancer remains one of the few examples of AI-based genetic biomarker detection from WSIs that has been approved for routine clinical use.^41^, ^42^, ^43^ *BRAF*, *TP53*, *KRAS*, and *PIK3CA* driver mutations were also included as they have been previously shown to be predictable from WSIs.^35^^,^^36^ Driver mutations were defined as being annotated as “Oncogenic” or “Likely Oncogenic” in OncoKB.^44^ For primary therapy outcome success in TCGA-STAD, patients were classified as responders if primary therapy outcome success was indicated as “Complete Remission/Response“. Conversely, patients with an outcome of “Progressive Disease” were considered non-responders. Patients indicated with “Stable Disease” or “Partial Response” were not included in the analysis of treatment response to improve predictive power by focusing on extreme outcomes.

### WSI preprocessing and feature extraction

WSI processing can be described in two broad steps; preprocessing and feature extraction (Fig. 1c). WSIs were first tessellated at 512×512 px at a resolution of 0.5 μm per pixel. Background and blurry patches were identified using Canny edge detection implemented with the *OpenCV* Python package.^45^^,^^46^ Patches found to contain less than two edges were considered as background and rejected from the dataset.^47^ Three feature extraction models were used to generate patch embeddings from both raw and stain normalized WSI patches (Fig. 1c). Stain normalization was carried out using the Macenko method.^12^ The first feature extractor consisted of a ResNet50 CNN pretrained on Imagenet (INPT-ResNet50). Model weights for INPT-ResNet50 were retrieved from the *torchvision.models* subpackage of the *PyTorch* deep learning library.^48^ The second feature extractor consisted of a ResNet50 CNN pretrained by Wang et al. on two large-scale histology datasets using a custom form of SSL, based on MoCo, termed clustering-guided contrastive learning (CCL-ResNet50).^22^^,^^49^ The model weights for CCL-ResNet50 were obtained from https://github.com/Xiyue-Wang/RetCCL. Both of the ResNet50-based feature extractors were used to extract *N*×2048 features per WSI where *N* is the total number of patches in the WSI. The third feature extractor consisted of a hybrid transformer–CNN architecture developed by Wang et al. (CTransPath).^21^ CTransPath was pretrained on massively unlabeled histology data using semantically relevant contrastive learning, a form of SSL based on MoCov3, and has been reported to be more robust and transferable than ImageNet pretraining and other SSL methods for computational pathology tasks.^16^^,^^50^ The model weights for CTransPath were obtained from https://github.com/Xiyue-Wang/TransPath. CTransPath was used to extract *N*×768 features per WSI. All feature extraction models were implemented using the *PyTorch* library.^48^

### ComBat batch correction

To estimate batch effects using an empirical Bayes framework, ComBat assumes that, for batch *i* and feature *g*, the additive batch effects γ_*ig*_, are drawn from a normal distribution prior with batch-specific mean μ_*i*_ and variance $\tau_{i}^{2}$ (i.e. $\gamma_{\mathrm{ig}}∼N\left(\mu_{i},\tau_{i}^{2}\right)$).^34^ Meanwhile, the multiplicative batch effects, δ_*ig*_, are drawn from an inverse gamma distribution prior with batch-specific shape parameter λ_*i*_ and scale parameter $\Theta_{i}$ (i.e. $\delta_{\mathrm{ig}}∼\mathrm{InverseGamma}\left(\lambda_{i},\Theta_{i}\right)$). The hyperparameters for the prior distributions, μ_*i*_, $\tau_{i}^{2}$, λ_*i*_, and $\Theta_{i}$, are estimated empirically via the method of moments.^34^

ComBat defines a location and scale model (Eq. 1) to center and standardize the variance of each batch for each feature independently.

(1) $$Y_{\mathrm{ijg}}=\alpha_{g}+\gamma_{\mathrm{ig}}+\delta_{\mathrm{ig}}\varepsilon_{\mathrm{ijg}}$$

In Eq. 1, $Y_{\mathrm{ijg}}$ represents the observed value of feature g in sample j from batch i. $\alpha_{g}$ corresponds to the average value of feature g across all batches, serving as a baseline. $\gamma_{\mathrm{ig}}$ and $\delta_{\mathrm{ig}}$ are the additive and multiplicative batch effects affecting feature g in batch i. Lastly, $\varepsilon_{\mathrm{ijg}}$ is the error term.

ComBat assumes that the errors are normally distributed (i.e., $\varepsilon_{\mathrm{ijg}}∼N\left(0,\sigma_{g}^{2}\right)$). The residuals of this model, which represent the feature values after accounting for the effects of batch, are adjusted using an empirical Bayes approach to systematically remove batch effects across all features (Eq. 2).

(2) $$Y_{\mathrm{ijg}}^{*}=\frac{Y_{\mathrm{ijg}}-{\hat{\alpha }}_{g}-{\hat{\gamma }}_{\mathrm{ig}}}{{\hat{\delta }}_{\mathrm{ig}}}+{\hat{\alpha }}_{g}$$

In Eq. 2, $Y_{\mathrm{ijg}}^{*}$ corresponds to the batch-corrected value for feature g in sample j from batch i. The parameters ${\hat{\alpha }}_{g}$, ${\hat{\gamma }}_{\mathrm{ig}}$, and ${\hat{\delta }}_{\mathrm{ig}}$ are empirical Bayes estimates for $\alpha_{g}$, $\gamma_{\mathrm{ig}}$, and $\delta_{\mathrm{ig}}$ respectively. Here, ${\hat{\alpha }}_{g}$ corresponds to the least-squares estimate of the overall mean of feature g, serving as the baseline around which corrections are applied. ${\hat{\gamma }}_{\mathrm{ig}}$ and ${\hat{\delta }}_{\mathrm{ig}}$ correct for additive and multiplicative batch effect, respectively.

ComBat harmonization was applied to raw patch embeddings from WSIs in the TCGA-COAD and TCGA-STAD training datasets independently. TSS was provided to ComBat as the batch argument. Deep features with 0 variance across all patches in a WSI were dropped from the dataset to ensure the proper functioning of ComBat. ComBat was implemented using the *PyCombat* Python package.^51^ For inference, the external data were standardized by providing *PyCombat* with the batch-corrected training set as a reference batch, ensuring that the external data conformed to the harmonized feature space.^52^

### Attention-based multiple instance learning

Attention-based multiple instance learning (A-MIL) models were trained on the obtained patch embeddings to predict clinical attributes from patch embeddings.^53^ Before passing feature vectors to the attention module, a single fully connected layer followed by ReLU activation was employed to embed K×2048 features to output 256-dimensional vectors $h_{k}$ for each patch k, where K, set to 512, was the maximum number of patches randomly sampled per patient at each epoch. The resulting feature vectors $h_{k}$ were then passed to an attention module to determine the attention score $a_{k}$ for each patch using Eq. 3, where $h\in R^{256}$, $V\in R^{128\times 256}$, $w\in R^{128\times 1}$. The attention module consisted of a fully connected linear layer with tanh activation, followed by a second fully connected linear layer with softmax activation, resulting in an K×1 attention score vector.

(3) $$a_{k}=\frac{e^{W^{T}\tanh\left({\mathrm{Vh}}_{k}\right)}}{\sum_{j=1}^{K}e^{W^{T}\tanh\left({\mathrm{Vh}}_{j}\right)}}$$

The resulting attention score vector, $a_{k}$, was used to weight the K×256 patch-level feature vectors, $h_{i}$, resulting in a 1×256 feature vector $h_{\mathrm{sum}}$ per WSI (Eq. 4).

(4) $$h_{\mathrm{sum}}=\sum_{i=1}^{K}a_{i}h_{i}$$

The final classification was achieved by passing the batch of $h_{\mathrm{sum}}$ vectors through a batch normalization layer and a dropout layer with probability set to 0.5. The resulting output was finally passed to a fully connected layer with softmax activation to obtain predicted probabilities for each class. The loss function was defined as the cross-entropy loss weighted inversely to the occurrence of classes. Models were trained with a batch size of 64 for a maximum of 64 epochs with early stopping if no reduction in the validation loss occurred for 16 epochs. The learning rate was varied according to the one cycle policy which schedules the learning rate with a cosine annealing with the maximum learning rate set to $1\times 10^{-4}$_._^54^ A-MIL models were trained to predict TSS and clinical attributes using raw, Macenko normalized, and ComBat harmonized patch embeddings. For predicting TSS, samples originating from TSSs with fewer than five samples were excluded to ensure that at least one sample from each TSS was present in each fold of 5-fold cross-validation experiments.

### Statistics and model evaluation

The performance of A-MIL models was evaluated using the mean area under the receiver operating characteristic curve (AUROC) across all folds for each cross-validation experiment. For multi-class classification experiments (i.e., predicting TSS and race), the mean one-vs-rest (OvR) AUROC across all folds in an experiment was used as the primary statistical endpoint. Comparisons between the mean AUROC values of separate experiments were carried out using a two-sided paired *t*-tests with an FDR of 0.05. To assess if a resulting mean AUROC was significantly greater than random chance (i.e. AUROC = 0.5), one-sided *t*-tests were performed. A 5-fold cross-validation was employed for training all models with the exception of the model trained to predict primary therapy outcome success, where 3-fold cross-validation was used due to the lower number of samples available. To evaluate the generalizability of A-MIL models trained using ComBat harmonized features, models were trained on the entire TCGA-COAD dataset and deployed on CPTAC-COAD (Fig. 1d). Confidence intervals for externally validated models were computed using 10× bootstrapping. Statistics were computed using the *scikit-learn* and *scipy* Python packages.^55^^,^^56^

### Associating batch corrections with visual WSI features

The degree of batch correction applied to each patch embedding was measured by taking the Euclidean distance between pairs of raw and ComBat harmonized embeddings. To visualize the batch correction applied across WSI patches, heatmaps were generated using calculated Euclidean distances. The Euclidean distances were normalized within each batch of samples to enable comparison between WSIs originating from the same TSS. Heatmaps were generated such that patches with greater distances between the raw and corrected embeddings were highlighted by higher intensity values on the heatmap, thus providing a visual representation of the extent of batch correction across the WSI. Euclidean distance measures and heatmaps were generated using *numpy*, *matplotlib*, and *seaborn* Python packages.^57^, ^58^, ^59^

## Results

### Cohort characteristics

The TCGA-COAD and TCGA-STAD datasets were used as primary discovery cohorts for this study. The TCGA-STAD cohort comprised 373 patients with WSIs. Meanwhile, the TCGA-COAD cohort consisted of 434 patients with WSIs available. For predicting TSS, samples originating from TSSs with fewer than five samples were excluded from the analysis. This resulted in 422 samples from 14 unique TSSs in TCGA-COAD and 357 samples from 12 unique TSSs in TCGA-STAD for the analysis. The CPTAC-COAD cohort used for external validation consisted of 104 patients with WSIs available. Clinicopathological characteristics for all cohorts used in this study are shown in Table 1.

**Table 1:** Clinicopathological characteristics for patients in the TCGA-STAD and TCGA-COAD training cohorts and the CPTAC-COAD external validation cohort.

Characteristic	TCGA-STAD			TCGA-COAD			CPTAC-COAD		
	*N*	%	% Missing	*N*	%	% Missing	*N*	%	% Missing
**Age**			1.07%			4.38%			1.92%
>65	197	52.82%		242	55.76%		52	50.00%	
≤65	172	46.11%		173	39.86%		50	48.08%	
**Sex**			0%			0.23%			0%
Male	246	65.95%		222	51.27%		41	39.42%	
Female	127	34.05%		211	48.73%		63	60.58%	
**Stage**			2.15%			2.77%			0%
Stage I	47	12.60%		73	16.86%		12	11.54%	
Stage II	115	30.83%		164	37.88%		42	40.39%	
Stage III	169	45.31%		125	28.87%		43	41.35%	
Stage IV	34	9.11%		60	13.86%		7	6.73%	
**Race**			12.00%			36.41%			100%
White	234	62.40%		207	75.00%		NA	NA	
Black	13	3.47%		58	21.01%		NA	NA	
Asian	83	22.12%		11	3.99%		NA	NA	
**MSI status**			12.33%			26.27%			0%
MSS	228	61.13%		207	64.69%		80	76.92%	
MSI	99	26.54%		113	35.31%		24	23.08%	
***TP53* driver**			0%			4.38%			0%
No driver	197	52.82%		212	51.08%		56	53.85%	
Driver	176	47.18%		203	48.92%		48	46.15%	
***PIK3CA* driver**			0%			4.38%			NI
No driver	319	85.52%		366	88.19%		NI	NI	
Driver	54	14.48%		49	11.81%		NI	NI	
***BRAF* driver**			NI			4.38%			0%
No driver	NI	NI		49	84.33%		89	85.58%	
Driver	NI	NI		366	11.29%		15	14.42%	
***KRAS* driver**			NI			4.38%			0%
No driver	NI	NI		257	61.93%		70	67.31%	
Driver	NI	NI		158	38.07%		34	32.69%	
***RNF43* driver**			NI			4.38%			
No driver	NI	NI		370	89.16%		NI	NI	
Driver	NI	NI		45	10.84%		NI	NI	
***KMT2B* mutated**			0%			4.38%			0%
Wild-type	340	91.15%		370	89.16%		88	84.62%	
Mutated	33	8.85%		45	10.84%		16	15.38%	

**Primary therapy**									
**Outcome success**			51.47%			NI			NI
Complete response	129	70.88%		NI	NI		NI	NI	
Progressive disease	42	23.08%		NI	NI		NI	NI	

**NI**: Not included. **NA**: Not available.

### Selecting clinical attributes in TCGA

#### TCGA-COAD

For TCGA-COAD, five attributes with at least 30 samples in the minority class were found to be associated with TSS and were therefore included for analysis. These included race ($\chi^{2}=386.2$; $P=3.04\times 10^{-56}$), *TP53* driver mutations ($\chi^{2}=49.9$; $P=9.48\times 10^{-4}$), *KRAS* driver mutations ($\chi^{2}=55.7$; $P=9.48\times 10^{-4}$), *RNF43* driver mutations ($\chi^{2}=57.52$; $P=8.65\times 10^{-5}$), and *KMT2B* mutations ($\chi^{2}=57.91$; $P=7.63\times 10^{-5}$). Additionally, MSI status ($\chi^{2}=26.2$; $P=2.44\times 10^{-1}$) and *BRAF* driver mutations ($\chi^{2}=29.8$; $P=1.55\times 10^{-1}$) were included as they have been previously shown to be predictable from WSIs in COAD.^35^^,^^41^

#### TCGA-STAD

For TCGA-STAD, two attributes with at least 30 samples in the minority class were found to be associated with TSS and were therefore included for analysis. These included race ($\chi^{2}=289.47$; $P=1.92\times 10^{-40}$) and primary therapy outcome success ($\chi^{2}=52.6$; $P=2.24\times 10^{-6}$). Other putative predictable biomarkers that were not found to be associated with TSS but were included for the analysis consisted of MSI status ($\chi^{2}=25.7$; $P=1.76\times 10^{-1}$), *TP53* driver mutations ($\chi^{2}=21.04$; $P=4.56\times 10^{-1}$), *PIK3CA* driver mutations ($\chi^{2}=15.86$; $P=7.78\times 10^{-1}$), and *KMT2B* driver mutations ($\chi^{2}=17.23$; $P=6.97\times 10^{-1}$).^35^^,^^40^ This yielded a total of six clinical attributes that were included to be predicted from WSIs in the TCGA-STAD dataset. A full list of the included biomarkers along with results of chi-square tests against TSS is shown in Supplementary Table 1.

### ComBat harmonization can effectively reduce site-specific information in deep histology features

**Fig. 2:**
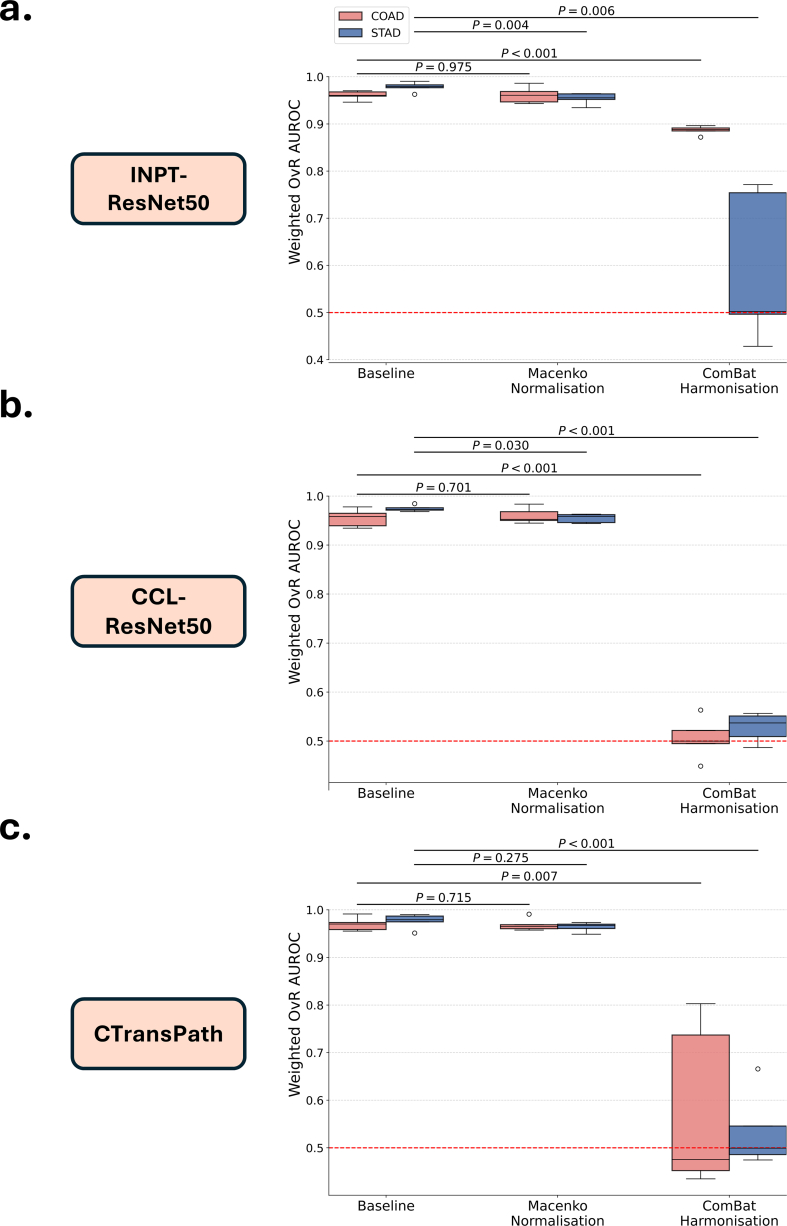
Effect of ComBat harmonization on A-MIL model performance at predicting TSS. Boxplots represent the distribution of OvR AUROC across each cross-validation fold. (a) Predicting TSS using INPT-ResNet50 features in TCGA-COAD (red) and TCGA-STAD (blue). (b) Predicting TSS using CCL-ResNet50 features in TCGA-COAD and TCGA-STAD. (c) Predicting TSS using CTransPath features in TCGA-COAD and TCGA-STAD. (For interpretation of the references to color in this figure legend, the reader is referred to the web version of this article.)

Five-fold cross-validation was used to assess the performance of A-MIL models at predicting TSS in selected TCGA datasets. The primary statistical endpoint was the mean OvR AUROC, weighted by the number of true instances for each label. Without implementing any batch effect mitigation methods, the models achieved a mean OvR AUROC of over 0.95 across all three feature extractors in both the TCGA-COAD and TCGA-STAD cohorts (Table 2). Applying Macenko color normalization to the extracted patches had a minimal impact on the models' performance in predicting TSS. However, when ComBat harmonization was applied to the extracted histology features, a noticeable decline in mean OvR AUROC was observed across all three feature extractors (Fig. 2:2). In five out of the six combinations of cancer type and feature extractor, ComBat harmonization reduced the model performance at predicting TSS to a level no greater than random chance. Notably, ComBat harmonization of features extracted using INPT-ResNet50 in the TCGA-COAD dataset (Fig. 2:.2a) only resulted in a modest decrease in performance at distinguishing TSS (T=−14.665; $P=1.26\times 10^{-4}$), indicating that TSS-specific information remained embedded in the extracted features (mean OvR AUROC = 0.887). To further explore the effects of different types of batch corrections, ComBat was applied separately for additive and multiplicative batch effects. The results demonstrate that correcting for additive batch effects led to a more pronounced reduction in model performance at predicting TSS compared to correcting for multiplicative effects (Supplementary Table 2).

**Table 2:** A-MIL model performance at predicting TSS in COAD and STAD datasets. Model performance is compared using different feature extractors and batch effect mitigation methods.

Dataset	Feature extractor	Mitigation method	Mean AUROC (Range)	*t*-Statistic vs. Baseline	*p*-Value vs. Baseline	*t*-Statistic > 0.5	*p*-Value > 0.5
**COAD**		Baseline	0.961 (0.946–0.970)	–	–	56.548	2.93×10^-7^
	INPT-ResNet50	Macenko	0.961 (0.943–0.986)	0.034	0.975	59.021	2.47×10^-7^
		ComBat	0.887 (0.872–0.896)	−14.665	1.26×10^-4^	94.272	3.80×10^-8^
		Baseline	0.955 (0.935–0.978)	–	–	56.548	2.93×10^-7^
	CCL-ResNet50	Macenko	0.960 (0.945–0.960)	0.413	0.701	64.889	1.69×10^-7^
		ComBat	0.506 (0.449–0.563)	−24.642	1.61×10^-5^	0.31	0.386
		Baseline	0.970 (0.955–0.991)	–	–	73.632	1.01×10^-7^
	CTransPath	Macenko	0.969 (0.958–0.991)	−0.393	0.715	78.746	7.79×10^-8^
		ComBat	0.580 (0.435–0.803)	−5.151	6.74×10^-3^	1.027	0.181
**STAD**		Baseline	0.978 (0.963–0.990)	–	–	105.622	2.41×10^-8^
	INPT-ResNet50	Macenko	0.954 (0.935–0.964)	−5.993	3.90×10^-3^	83.968	6.03×10^-8^
		ComBat	0.591 (0.428–0.771)	−5.212	6.46×10^-3^	1.265	0.137
		Baseline	0.975 (0.968–0.984)	–	–	170.703	3.53×10^-9^
	CCL-ResNet50	Macenko	0.955 (0.944–0.963)	−3.289	0.030	111.557	1.94×10^-8^
		ComBat	0.528 (0.487–0.557)	−29.517	7.84×10^-6^	2.142	0.049
		Baseline	0.977 (0.951–0.990)	–	–	69.428	1.29×10^-7^
	CTransPath	Macenko	0.964 (0.949–0.974)	−1.262	0.275	107.593	2.24×10^-8^
		ComBat	0.534 (0.475–0.666)	−10.788	4.19×10^-4^	0.978	0.192

### Effect of ComBat harmonization on deep histology features for predicting clinical attributes

Deep histology features that can effectively differentiate TSSs provide an avenue for deep learning models aimed at predicting clinical attributes to instead learn confounding site-specific features. To demonstrate that ComBat batch correction of effectively mitigate this risk, the performance of A-MIL models at predicting various clinical attributes trained with unharmonized and ComBat-harmonized features was compared.

#### Clinical attributes associated with tissue-source site

Race was found to be highly associated with TSS in both the TCGA-COAD ($P=3.04\times 10^{-54}$) and TCGA-STAD ($P=1.92\times 10^{-40}$) and could accurately be predicted when training an A-MIL model on histology features extracted using any of the three feature extractors (Table 3, Supplementary Table 3). A marked reduction in performance at predicting race was observed after ComBat harmonization was applied to CTransPath-extracted histology features in both the TCGA-COAD (Δ mean OvR AUROC =−0.364) and TCGA-STAD datasets (Δ mean OvR AUROC =−0.186). A similar reduction in performance at predicting race was observed when using the CCL-ResNet50 extracted histology features, however, no reduction was noted when applying ComBat harmonization to histology features extracted using INPT-ResNet50 (Supplementary Table 3).

**Table 3:** Impact of ComBat harmonization at predicting selected targets in TCGA-COAD. Comparison of A-MIL model performance according to AUROC trained using both unharmonized (Pre-ComBat) and harmonized (Post-ComBat) CTransPath extracted features.

Target	Pre-ComBat		Post-ComBat		Pre-Combat vs. Post-Combat
	Mean AUROC	*p*-Value	Mean AUROC	*p*-Value	*p*-Value
	(Range)	AUROC > 0.5	(Range)	AUROC > 0.5	
Race	0.835 (0.758–0.874)	3.95×10^-5^	0.471 (0.419–0.530)	8.96×10^-1^	6.94×10^-5^
*BRAF* driver	0.728 (0.648–0.805)	7.54×10^-4^	0.741 (0.664–0.796)	3.30×10^-4^	3.74×10^-1^
*TP53* driver	0.686 (0.592–0.785)	2.15×10^-3^	0.680 (0.616–0.760)	9.91×10^-4^	7.59×10^-1^
*KMT2B* mutation	0.681 (0.619–0.757)	1.38×10^-3^	0.639 (0.587–0.703)	1.21×10^-3^	1.80×10^-1^
*RNF43* driver	0.642 (0.502–0.818)	2.69×10^-2^	0.538 (0.374–0.626)	2.30×10^-1^	2.18×10^-1^
MSI status	0.667 (0.515–0.741)	6.60×10^-3^	0.669 (0.639–0.737)	3.40×10^-4^	9.52×10^-1^
*KRAS* driver	0.598 (0.512–0.663)	8.62×10^-3^	0.521 (0.434–0.617)	2.94×10^-1^	7.21×10^-2^

**Fig. 3:**
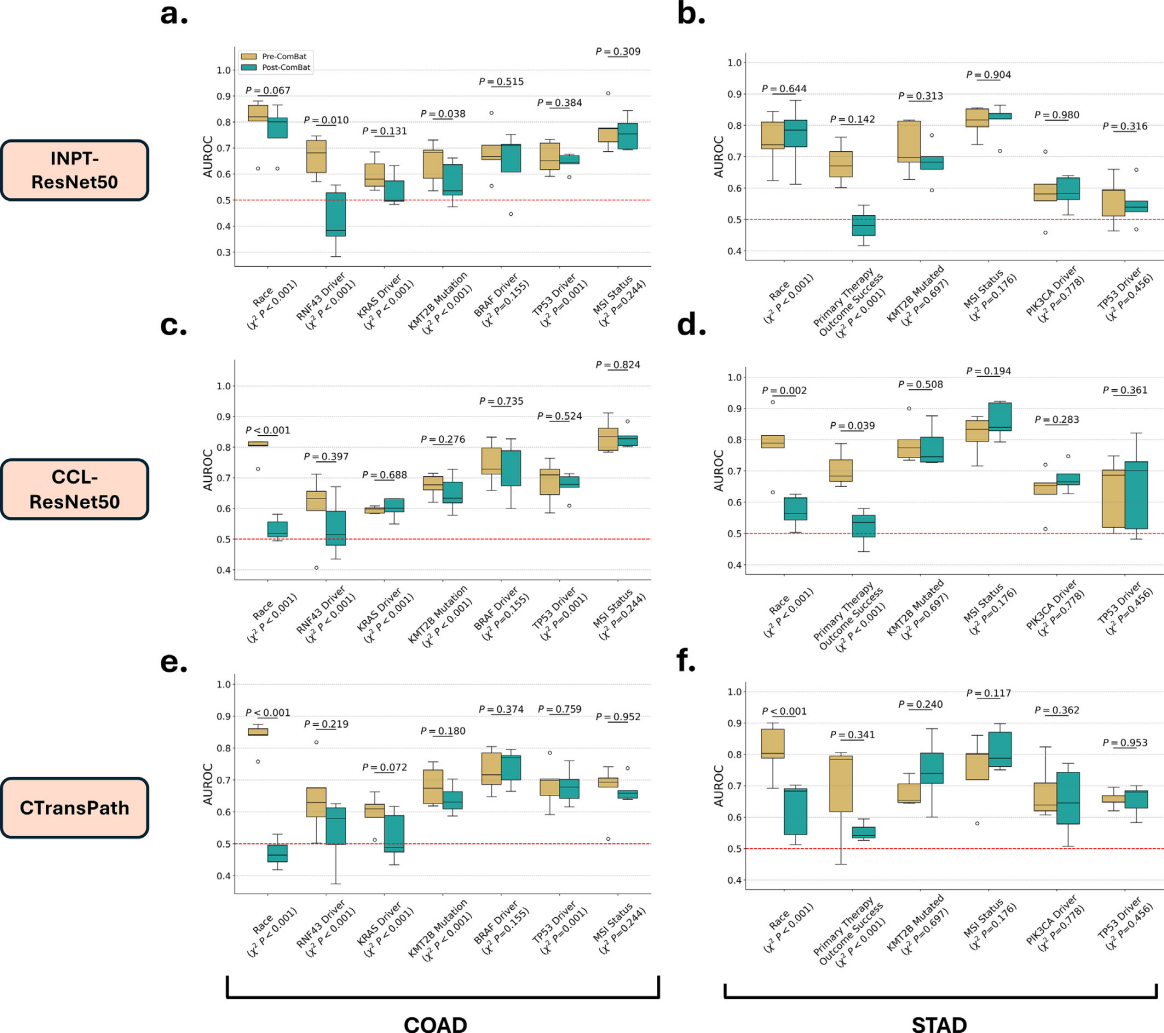
Effect of ComBat harmonization on A-MIL model performance at predicting clinical attributes. Boxplots represent the distribution of AUROC across each cross-validation fold. (a) Predicting clinical attributes using INPT-ResNet50 features in TCGA-COAD. (b) Predicting clinical attributes using INPT-ResNet50 features in TCGA-STAD. (c) Predicting clinical attributes using CCL-ResNet50 features in TCGA-COAD. (d) Predicting clinical attributes using CCL-ResNet50 features in TCGA-STAD. (e) Predicting clinical attributes using CTransPath features in TCGA-COAD. (f) Predicting clinical attributes using CTransPath features in TCGA-COAD.

Other clinical attributes associated with TSS in TCGA-COAD included *TP53*, *KRAS*, and *RNF43* driver mutations, as well as *KMT2B* mutations. Both *TP53* driver mutations and *KMT2B* mutations remained predictable after applying ComBat harmonization to extracted histology features. Meanwhile, the performance of A-MIL models at predicting *RNF43* and *KRAS* driver mutations decreased to no greater than random chance after applying ComBat harmonization to the CTransPath-derived histology features (Fig. 3e; Table 3). This trend was also observed for *RNF43* mutations using the INPT-ResNet50 and CCL-ResNet50 feature extractors (Fig. 3a & c; Supplementary Table 3). Meanwhile only a minor reduction in mean AUROC was observed for *KRAS* driver mutations when using the INPT-ResNet50 and CCL-ResNet50 feature extractors (Fig. 3a & c; Supplementary Table 3).

In TCGA-STAD, primary therapy outcome success was the only included clinical attribute, other than race, that was found to be associated with TSS. Primary therapy outcome success could be predicted with a mean AUROC of 0.680 when training the A-MIL model with raw CTransPath-derived histology features, with a maximum AUROC across folds of 0.806 (Table 4). After applying ComBat harmonization to CTransPath-derived histology features, the mean AUROC was reduced to 0.554, whereas the maximum AUROC across folds was 0.595. This trend was also observed when using INPT-ResNet50 and CCL-ResNet50 derived histology features (Fig. 3; Supplementary Table 4).

**Table 4:** Impact of ComBat harmonization at predicting selected targets in TCGA-STAD. Comparison of A-MIL model performance according to AUROC trained using both unharmonized (Pre-ComBat) and harmonized (Post-ComBat) CTransPath extracted features.

Target	Pre-ComBat		Post-ComBat		Pre-Combat vs. Post-Combat
	Mean AUROC	*p*-Value	Mean AUROC	*p*-Value	*p*-Value
	(Range)	AUROC > 0.5	(Range)	AUROC > 0.5	
Race	0.813 (0.692–0.900)	5.37×10^-4^	0.627 (0.512–0.702)	1.76×10^-2^	7.09×10^-4^
MSI status	0.753 (0.580–0.861)	3.26×10^-3^	0.814 (0.751–0.898)	2.27×10^-4^	1.17×10^-1^
*PIK3CA* driver mutation	0.680 (0.608–0.824)	5.42×10^-3^	0.649 (0.507–0.771)	1.97×10^-2^	3.62×10^-1^
Primary therapy outcome success	0.680 (0.450–0.806)	1.29×10^-1^	0.554 (0.527–0.595)	5.98×10^-2^	3.41×10^-1^
*KMT2B* mutation	0.678 (0.645–0.739)	3.56×10^-4^	0.747 (0.600–0.882)	3.20×10^-3^	2.40×10^-1^
*TP53* driver mutation	0.656 (0.621–0.695)	1.13×10^-4^	0.655 (0.583–0.700)	1.01×10^-3^	9.42×10^-1^

#### Putative predictable clinical attributes independent of TSS

MSI status and *BRAF* driver mutations have both been previously identified as predictable from WSIs in COAD.^35^ ComBat harmonization had no impact on the performance of A-MIL models at predicting MSI across all three feature extractor models (Table 3; Supplementary Table 3). Similarly, A-MIL model performance at predicting *BRAF* driver mutations was unaffected by applying ComBat harmonization to features extracted using either of the three feature extraction models (Table 3, Supplementary Table 3).

MSI status, *PIK3CA* and *TP53* driver mutations, as well as *KMT2B* mutations have all been previously reported as predictable from WSIs in STAD.^35^ As per COAD, it was found that ComBat harmonization of deep learning-derived histology features had no impact on the performance of A-MIL models at predicting these clinical attributes (Table 4; Supplementary Table 4).

### Generalizability of ComBat harmonized deep features

The generalizability of A-MIL models trained with unharmonized and ComBat harmonized CCL-ResNet50 derived features was compared for selected clinical attributes in COAD. Models trained to predict *BRAF*, *TP53*, and *KRAS* driver mutations as well as MSI status using TCGA-COAD data were evaluated on the CPTAC-COAD cohort. Models trained to predict MSI status and *BRAF* driver mutations exhibited robust performance in the external dataset using both unharmonized and ComBat harmonized features. A slight increase in mean AUROC was observed in predicting *BRAF* driver mutations after applying ComBat harmonization to the CCL-ReseNet50 derived features. However, when trained to predict MSI, ComBat harmonization resulted in a slight decrease in performance in the external cohort (Table 5). The A-MIL model trained to predict *TP53* driver mutations failed to generalize to the CPTAC-COAD cohort using either unharmonized or ComBat harmonized features. Meanwhile, a decrease in performance was observed in the external AUROC when predicting *KRAS* driver mutations (Table 5). Similar trends in performance were observed when using non-parametric priors during ComBat harmonization (Supplementary Table 5).

**Table 5:** Generalizability of A-MIL models trained using TCGA-COAD (internal) CCL-ResNet50 features and validated on CPTAC-COAD (external).

Target	Internal Mean AUROC ± Std		External AUROC ± Conf. Int.	
	Pre-ComBat	Post-ComBat	Pre-ComBat	Post-ComBat
MSI	0.830 **±** 0.03	0.840 **±** 0.05	0.853 **±** 0.12	0.815 **±** 0.11
*BRAF* driver	0.720 **±** 0.07	0.740 **±** 0.10	0.622 **±** 0.18	0.691 **±** 0.14
*TP53* driver	0.687 **±** 0.07	0.675 **±** 0.04	0.486 **±** 0.11	0.500 **±** 0.11
*KRAS* driver	0.595 **±** 0.01	0.601 **±** 0.03	0.603 **±** 0.12	0.522 **±** 0.12

**Std.**: Standard Deviation; **Conf. Int.**: 95% Confidence Interval Range.

### Visualization of batch corrections on WSIs

**Fig. 4:**
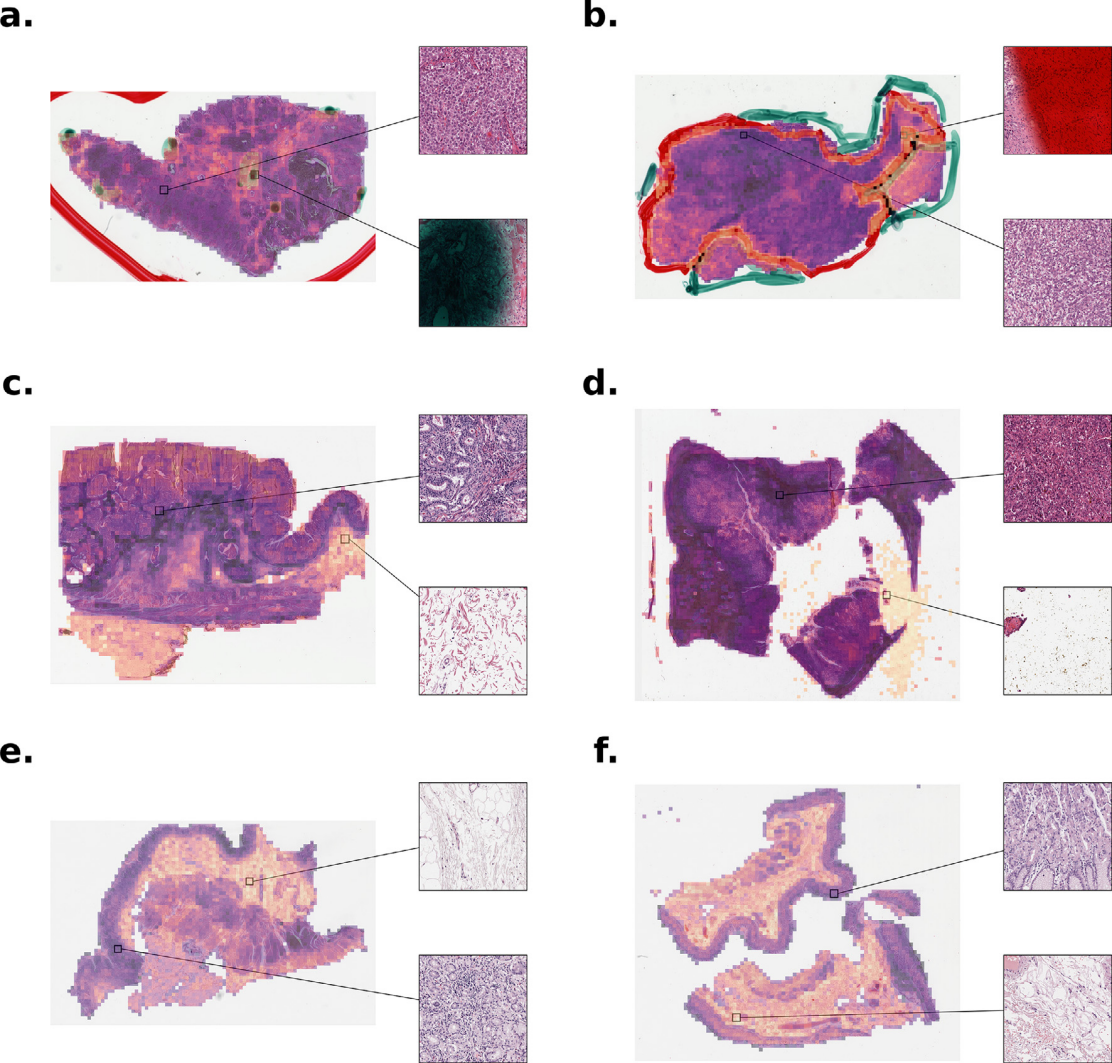
Heatmaps of batch corrected WSI regions using ComBat. (a) Patient TCGA-BR-8295 from Asterand TSS. (b) Patient TCGA-BR-7957 from Asterand TSS. (c) Patient TCGA-VQ-AA69 from Barretos Cancer Hospital TSS. (d) Patient TCGA-VQ-A8PO from Barretos Cancer Hospital TSS. (e) Patient TCGA-D7-A74A from Greater Poland Cancer Center TSS. (f) Patient TCGA-D7-A6EX from Greater Poland Cancer Center TSS.

Regions of WSIs undergoing significant batch correction were visualized using heatmaps (Fig. 4). Heatmaps from the Asterand TSS revealed that areas with pen markings were subject to substantial correction by ComBat (Fig. 4a and b). WSIs from the Barretos Cancer Hospital highlighted tissue folds, background patches with debris, and adipose tissue undergoing greater correction, whereas tumor regions exhibited comparatively lower correction (Fig. 4c and d). Meanwhile, WSIs from the Greater Poland Cancer Center appeared to be corrected for loose mixed connective tissue of the lamina propria (Fig. 4e and f).

## Discussion

In this study, we evaluated the effectiveness of ComBat batch correction to mitigate site-specific confounding features in deep learning-derived histology features. We observed that TSS could be accurately predicted from WSIs using various feature extractor models. However, the application of ComBat harmonization could significantly reduce the predictability of TSS, demonstrating its potential to eliminate site-specific biases.

### ComBat harmonization of deep features effectively reduces the impact of confounding batch effects in computational pathology

WSIs are prone to a variety of technical biases arising from processes such as tissue preparation, scanning, and storage. These biases tend to cluster by clinical center, or TSS, and can lead to AI models inadvertently learning technical artefacts instead of true histological information. Information relating to TSS was encoded in the patch embeddings obtained from all three feature extractors, such that TSS could be predicted with high performance (mean OvR AUROC >0.95), even after applying Macenko normalization. However, ComBat harmonization of patch embeddings obtained with SSL-based feature extractors effectively reduced the predictability of TSS. This finding demonstrates the potential of modeling, and correcting for batch effects in the latent space for computational pathology. The reduction in predictability of TSS from patch embeddings was less pronounced using INPT-ResNet50 when compared to the two SSL-based feature extractors, allowing attributes such as race to remain predictable even after applying ComBat harmonization to INPT-ResNet50 extracted histology features. Since ComBat is limited to correcting for additive and multiplicative batch effects in individual features, it could be hypothesized that Imagenet pretraining results in batch effects that are represented as nonlinear interactions between features, which are not adequately captured using ComBat. This is further discussed in ComBat harmonization of deep features effectively reduces the impact of confounding batch effects in computational pathology. A separate analysis of additive and multiplicative batch effects revealed that correcting for additive batch effects alone resulted in a reduction in TSS predictability comparable to that achieved when correcting for both additive and multiplicative effects. In contrast, correcting solely for multiplicative batch effects had a more moderate impact, with models retaining performance levels closer to the baseline. These findings suggest that additive batch effects may have a more pronounced role in deep learning-derived histology features than multiplicative batch effects.

A recent study by Vaidya et al. showed that AI model performance in computational pathology can vary widely by demographic groups such as race.^24^ Vaidya et al. proposed various racial bias mitigation strategies to improve model fairness, such as importance weighting and adversarial regularization to control for race. In our study, race was found to be highly associated with TSS in both TCGA cohorts and could be predicted with high accuracy from WSIs. However, combining SSL-based feature extractors with ComBat harmonization significantly reduced the predictability of race from patch embeddings (P<0.001), despite controlling for TSS instead of race. Making patch embeddings agnostic to race using ComBat may help mitigate demographic biases in model performance and could be a valuable avenue for future research.

End-to-end tasks, such as predicting treatment response, remain a challenge in computational pathology and continues to be an active area of research.^60^ In this study, we showed that A-MIL models trained using unharmonized patch embeddings achieved moderate performance at predicting primary therapy outcome success in TCGA-STAD. However, a reduction in performance was observed across all three feature extractors after applying ComBat harmonization, indicating that AI models were likely learning batch effects despite being trained to predict primary therapy outcome success. Additionally, in TCGA-COAD, ComBat harmonization resulted in a slight reduction for predicting *RNF43* and *KMT2B* mutations, both of which were found to be moderately associated with TSS and have been previously reported as predictable from WSIs.^35^ Whereas this reduction in performance was not found to be statistically significant, it suggests that AI models trained using unharmonized deep features might leverage both true histological signals and batch effects during training, leading to a slight inflation in model performance when evaluated through cross-validation. These findings highlight how AI models in computational pathology can utilize batch effects to seemingly learn clinically relevant tasks and how ComBat harmonization can effectively mitigate this risk.

WSI heatmaps were generated to gain insight into potential visual features for which ComBat aimed to correct. In the selected WSIs, such regions included pen-markings, background patches containing debris which passed through initial segmentation steps, and tissue folds. In contrast, a lower degree of correction was observed in tumor regions suggesting that biological signals are more stable across batches compared to artifacts introduced by tissue processing or WSI scanning. However, this analysis was limited to a small number of cases and TSSs, and it is possible that other batch effects corrected by ComBat were not captured in these samples or were not easily discernible to the human eye.

### ComBat batch correction retains clinically relevant histological signal in deep features

Whereas ComBat harmonization reduced the models' ability to learn batch effects, the prediction performance of clinically relevant genetic attributes remained consistent after applying ComBat, indicating that true histology information was preserved in the harmonized feature set.

Computational pathology researchers have focused extensively on MSI due to its high-level of predictability from WSIs.^41^^,^^61^ In our study, ComBat harmonization had no impact on the predictability of MSI from patch embeddings in either the COAD or STAD datasets, demonstrating that true histology information is retained when applying ComBat harmonization to extracted histology features. Similarly, the predictability of *TP53* driver mutation status remained consistent for given feature extractors after applying ComBat in both the STAD and COAD cohorts. In TCGA-STAD, the models' performance in predicting *PIK3CA* mutation status remained stable across all three feature extractors after applying ComBat. Likewise, in TCGA-COAD, the ability to predict *BRAF* driver mutations showed no change before and after ComBat harmonization across all feature extractors. Neither *PIK3CA* mutations in TCGA-STAD nor *BRAF* mutations in TCGA-COAD were found to be associated with TSS, demonstrating that that ComBat harmonization effectively preserves true histological signals.

To evaluate the generalizability of models trained with ComBat harmonized features, we deployed models trained with CCL-ResNet50 features to predict key clinical attributes in TCGA-COAD within the CPTAC-COAD cohort. No drop in performance in the external validation cohort was observed following utilization of the harmonized feature set for either MSI status or *BRAF* driver mutation status. The model trained to predict *TP53* driver mutations failed to generalize to the external cohort when using either raw or harmonized features for training. Meanwhile, a slight reduction in performance was observed when deploying the *KRAS* driver mutation model on the external data when ComBat harmonization was applied. These results indicate that whereas ComBat harmonization of deep features can retain true histological signal, the effectiveness of ComBat harmonization may vary depending on the specific genetic attribute being predicted.

### Limitations

Whereas we have shown that batch correction using ComBat is a promising technique to mitigate batch effects in digital pathology datasets, its application comes with certain limitations. Firstly, ComBat assumes that batch effects in the data are either additive or multiplicative, which allows for their estimation and subsequent correction. This assumption is based on the understanding that batch effects can manifest as systematic changes in the scale (multiplicative) or the offset (additive) of the feature values across different batches. Whereas this assumption may hold true in the case of RNA sequencing data, deep learning models are known to extract abstract feature representations that may not linearly correlate with visual features. This means that batch effects may exist in the interactions between deep features and could still be exploited by AI models after ComBat harmonization. Future work may explore alternative batch correction methods that can be employed in the latent space, such as the use of conditional variational autoencoders.^62^^,^^63^ Secondly, ComBat assumes that the underlying confounding features are adequately captured within the available batch labels. In some cases, the available batch information may not fully account for the diverse confounding features contributing to variations in the data. For example, a number of TSSs may use the same scanner or staining protocol, resulting in WSIs from different TSSs containing similar batch effects. Alternatively, information on TSS may not be readily available for all WSIs in a dataset. Future work may draw on clustering WSI datasets based on high-dimensional quality control metrics to assign batch labels instead of relying on TSS labels.^64^ Thirdly, our work is limited to two cancer types within publicly-available datasets. Further research is needed to validate these findings in a broader range on cancer types and larger datasets.

## Conclusion

Our findings demonstrate the necessity of rigorous batch correction techniques and external validation to ensure reliable and clinically applicable AI models. ComBat harmonization of deep learning-derived WSI features offers a promising solution to mitigate confounding site-specific batch effects in digital pathology datasets. This approach enables the reliable integration of digital pathology datasets from multiple sources. Future studies into the use of latent space batch correction in computational pathology across diverse clinical settings are required to further validate these findings.

## Funding sources

This research was funded by 10.13039/501100001602Science Foundation Ireland (SFI) through the SFI Centre for Research Training in Genomics Data Science under grant number 18/CRT/6214.

## Declaration of competing interest

The authors declare that they have no known competing financial interests or personal relationships that could have appeared to influence the work reported in this paper.
